# AI-Driven Framework for Enhanced and Automated Behavioral Analysis in Morris Water Maze Studies

**DOI:** 10.3390/s25051564

**Published:** 2025-03-04

**Authors:** István Lakatos, Gergő Bogacsovics, Attila Tiba, Dániel Priksz, Béla Juhász, Rita Erdélyi, Zsuzsa Berényi, Ildikó Bácskay, Dóra Ujvárosy, Balázs Harangi

**Affiliations:** 1 Faculty of Informatics, University of Debrecen, H-4028 Debrecen, Hungaryharangi.balazs@inf.unideb.hu (B.H.); 2 Department of Pharmacology and Pharmacotherapy, University of Debrecen, H-4032 Debrecen, Hungary; 3 Department of Emergency Medicine, University of Debrecen Clinical Centre, H-4032 Debrecen, Hungary; 4 Department of Dentistry, University of Debrecen, H-4032 Debrecen, Hungary

**Keywords:** morris water maze, convolutional neural network, machine learning, behavioral classification

## Abstract

The Morris Water Maze (MWM) is a widely used behavioral test to assess the spatial learning and memory of animals, particularly valuable in studying neurodegenerative disorders such as Alzheimer’s disease. Traditional methods for analyzing MWM experiments often face limitations in capturing the complexity of animal behaviors. In this study, we present a novel AI-based automated framework to process and evaluate MWM test videos, aiming to enhance behavioral analysis through machine learning. Our pipeline involves video preprocessing, animal detection using convolutional neural networks (CNNs), trajectory tracking, and postprocessing to derive detailed behavioral features. We propose concentric circle segmentation of the pool next to the quadrant-based division, and we extract 32 behavioral metrics for each zone, which are employed in classification tasks to differentiate between younger and older animals. Several machine learning classifiers, including random forest and neural networks, are evaluated, with feature selection techniques applied to improve the classification accuracy. Our results demonstrate a significant improvement in classification performance, particularly through the integration of feature sets derived from concentric zone analyses. This automated approach offers a robust solution for MWM data processing, providing enhanced precision and reliability, which is critical for the study of neurodegenerative disorders.

## 1. Introduction

Researching dementia and Alzheimer’s disease is paramount due to their widespread impact on global populations, presenting substantial challenges to healthcare infrastructures and familial support systems. The increasing prevalence of dementia, particularly among the elderly, emphasizes the importance of understanding its various manifestations and implications. A thorough investigation into the etiology and pathophysiology of these conditions holds the promise of developing early detection methods, optimizing therapeutic interventions, and potentially curative treatments. Rodent models serve as valuable tools for studying the biological mechanisms underlying dementia and Alzheimer’s disease. Longitudinal studies are made possible by mice and rats, which enable researchers to monitor changes in behavior, cognition, and brain pathology over time. This is crucial for comprehending the course of disease and assessing possible therapeutic interventions [1].

Several brain regions are involved in memory formation and retrieval. The hippocampus is a vital central nervous system (CNS) structure involved in memory formation, spatial navigation, and emotional regulation in mammals. Structurally, it consists of similar subfields and circuitry in rodents and humans, including the dentate gyrus, Cornus Ammonis-1 to Cornus Ammonis-3 (CA1-CA3) regions, and the subiculum. Despite differences in size and complexity, the fundamental principles governing hippocampal function and organization are highly conserved across species, making rodent models useful for investigating hippocampal-related disorders, including dementia and Alzheimer’s disease [2].

The Morris Water Maze (MWM) [3] offers a resilient and adaptable tool to explore spatial learning and memory within a controlled experimental environment, presenting valuable perspectives on cognitive mechanisms and brain functionality. During the MWM, animals navigate a pool of opaque water to locate a hidden platform using spatial cues or visual landmarks [4]. This navigational process entails the establishment of spatial representations and subsequent memory consolidation, both of which are functionalities closely linked to the hippocampus; thus, the MWM is considered a hippocampal-dependent task, where successful performance relies on the integrity and proper functioning of the hippocampus [5]. Various behavioral measures are recorded during the test, such as latency to find the platform, path taken to reach the platform, swimming speed, and strategies employed for navigation (e.g., direct swim, circling, and thigmotaxis). The amount of time the animal spends in each pool quadrant, the directionality, the latency to locate the platform in each trial, and the total distance swim in each trial are also common metrics [5].

These basic measurements and statistical analyses have faced considerable scientific scrutiny due to their limited ability to encompass the diverse array of animal behaviors observed in MWM experiments, as well as their inadequate correlation with the fundamental mechanisms underlying memory formation [6].

Over time, more advanced quantification methods have been proposed to enhance the characterization of behavior in the MWM, e.g., establishing scoring systems based on the time spent in different quadrants, or classifying different behavioral strategies of the rodents when searching for the platform [7]. Other researchers created a more comprehensive taxonomy of swimming paths, categorizing them into seven distinct types, or exploration strategies [8]. More recently, automated computer methods were applied to recognize animal trajectories and exploration strategies, based on single swimming path analyses [9]. The same group applied machine learning techniques to automatically detect animal behaviors, and they introduced an automated boosted classification approach utilizing majority voting, enhancing the accuracy of their classification method [6]. Utilizing computer-based learning algorithms facilitates the automated segregation of distinct animal cohorts (e.g., those with intact cognitive function, aged animals, and individuals with Alzheimer’s dementia), offering researchers valuable insights into hippocampal function. Nonetheless, this advancement necessitates a concomitant enhancement of these automated algorithms to bolster accuracy, thereby catering to the escalating demand for precise categorization in research endeavors.

**Figure 1:**
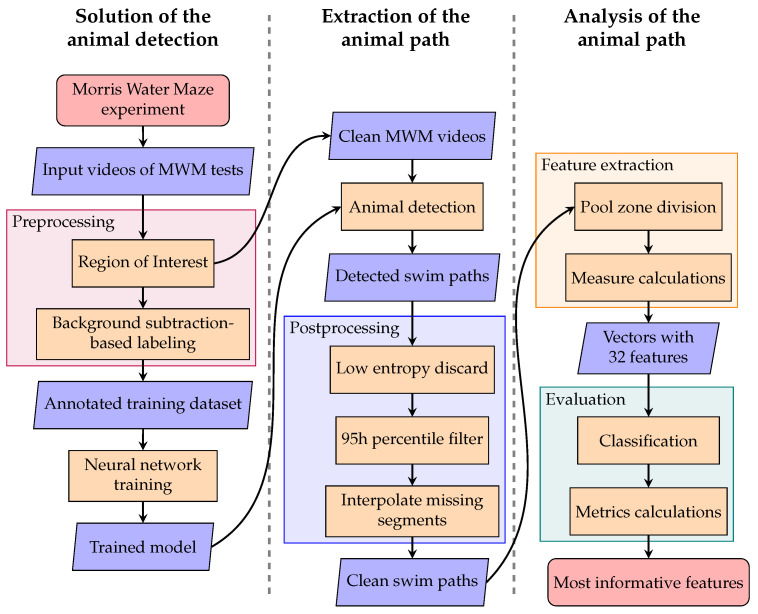
Flow chart of the MWM evaluation process.

In this paper, we introduce our AI-based framework developed for the automated processing of videos captured during the MWM trials. Our aim is to develop a fully automated pipeline that can be used by biology researchers for the evaluation of the recorded MWM-test videos using some enhanced machine learning-based tools. The main steps of the proposed pipelines can be seen in Figure 1. As a first step (see the first column on the left), we develop the appropriate machine learning-based algorithm to detect the animals in the pool during each frame of the input videos. In the next step (see the middle part), the process moves forward with the output of the trained model, which is able to detect animal positions with high accuracy, and in this way, we can track the movement of the animals. From this result, we construct the swimming paths of the animals, which still require some postprocessing steps. The output of this phase is the cleaned swimming paths, which is accurate enough to consider its main features. As a last step, we extract some meaningful descriptors of the tracked movements which could be used for a classification task. This final step is improved by considering our proposed features, which also improves the final classification accuracy and reliability.

The structure of the article also follows the outline presented in Figure 1: Section 2 details the originally created dataset as well as the methodologies used within each step of the process. In Section 2.1, the dataset of the MWM test is described, then in Section 2.2 all necessary preprocessing steps are given. In Section 2.3, we write about the method used for animal detection, while in Section 2.4, all the postprocessing steps are listed. The details of the feature extraction and the evaluation process are given in Section 2.5 and Section 2.6, respectively. Last, but not least, Section 3 contains the results of our analysis, and Section 4 concludes with some further discussion.

## 2. Materials and Methods

### 2.1. Data Overview

**Figure 2:**
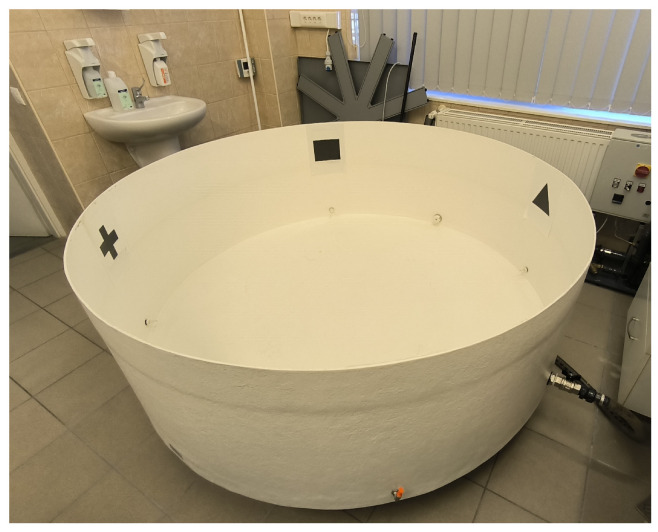
The experiment room with the pool setup.

During this study, the MWM tests were conducted at the Department of Pharmacology and Pharmacotherapy at the University of Debrecen, Hungary. The room with the experiment setup is shown in Figure 2. The circular pool at the center is 180 cm in diameter × 60 cm high, while the camera is situated above at 260 cm. The pool is filled with water (25 °C) to depths of 35 cm, and rendered opaque by added Acusol OP 301 opacifier (Fabricant Rohm&Haas, Landskrona, Sweden). A smaller circular platform is added to the pool for the test animals to escape from the water; this is invisible due to its height being 4 cm below the water surface. Regarding the size of the platform, the initial diameter is 10 cm, but we find that the rats often fall off because the older rats are significantly larger and heavier. As a result, we decide to increase the platform diameter to 14 cm to improve stability.

To aid the animal detection process, not only the pool water is dyed but also the test animals themselves in order to create greater contrast between the subject and the background. The room dedicated to the MWM tests contains wall mounts and posters serving as distal cues for the animals to learn. We pay attention such that the assistants carrying out the experiments (e.g., putting the rats in and out of the maze) always stand in the same place in the room, as they themselves also serve as distal cues. Additionally, as many protocols suggest, we also use pool cues that are four different markings (shapes) on the pool’s inner wall. These markings provide the reference points to separate the circular pool into four quadrants (as detailed in [3]).

The animals placed in the MWM test framework are male rats, from the Wistar strain. During the study, in total 74 rats are tested, from which 39 are between 12 and 16 weeks old, and 35 are between 24 and 28 months old. The rats receive 3 trials each day for 3 consecutive days, yielding daily averages for statistical analysis, not just the raw data of each separate trial. The test animals’ swimming are filmed to later analyze their cognitive abilities based on the paths they take while finding the platform. All of the videos that make up the dataset used in this study are created by the authors.

The dataset comprises 592 video files (444 × 444 resolution) of MWM tests. The animals are categorized according to their age, with 35 classified as older (2 years old or more) and 39 as younger. The videos can be divided into two categories: 251 videos (318,190 frames) feature older test animals, while 341 videos (412,249 frames) feature younger specimens. In this study, we focus our classification on the age of the animals. However, in the future, we intend to implement additional classification systems with the aim of evaluating the impact of various medical treatments.

### 2.2. Preprocessing

**Figure 3:**
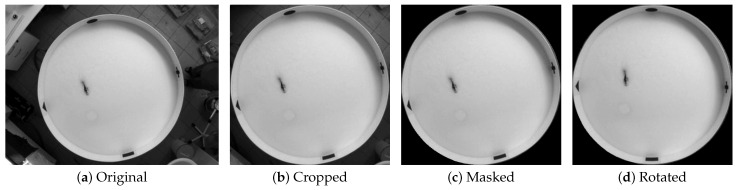
A frame of (**a**) the unprocessed raw video with the preprocessing steps: (**b**) cropping, (**c**) masking, and (**d**) rotating of the image.

First and foremost, it is a well-established practice to preprocess any raw dataset before applying models or utilizing it in any way. It is evident at first glance (see Figure 3a) that the captured video frames include not only the pool but parts of the surrounding room as well, which can be a distracting factor in the animal detection process. Moreover, the inconsistent placement between videos of the 4 shapes arranged on the inside wall of the pool can hinder later processing of the detected swimming path. These shapes define the 4 quadrants used in the evaluation steps, and thus their consistent position on every video and frame is crucial.

To achieve consistent positioning and to exclude the pool’s surroundings, we apply an automatic masking step to limit frames to focus only on the region of interest (ROI). Figure 3 depicts, from left to right, the original frame, then followed by the cropped image that only contains the bounding box of the pool. The third image is obtained by applying an automatic pattern recognition technique to mask out everything outside of the pool. In this step, we apply 2D convolution with a pre-made image used as a kernel containing only the pool border. This is used to search for the highest convolution value, indicating the best match to the original frame, finding the position of the pool, and centering it. Since the pool is not rotated properly yet, we keep rotating the frame until we find the best pattern match (also with 2D convolution with the same kernel) and thus obtain the right rotation angle for the frame so that the shapes at the edge of the pool are always in a consistent position on the preprocessed video, resulting in a masked and rotationally aligned image of the pool.

Before starting the process of detecting the animal on the videos with a complex convolutional neural network, first we need an already labeled training set of image frames, where the position of the animal is given by its bounding box. However, as annotating such a large amount of frames by hand is not a feasible route, we opt for the commonly used background subtraction-based method [10,11] as a semi-automated solution.

**Figure 4:**
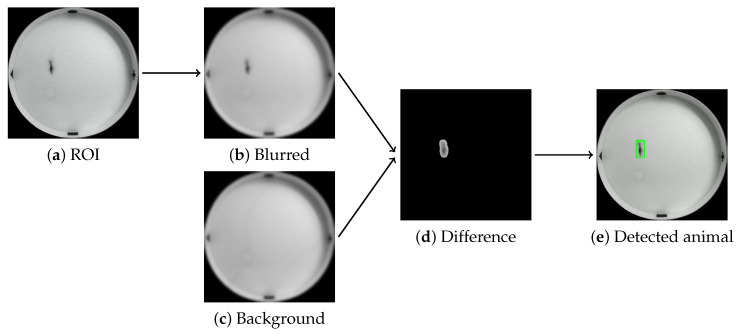
The process of detecting the animal in the image of the pool: (**a**) extracted ROI, (**b**) Gaussian blurred image, (**c**) precalculated comparing background, (**d**) differing image parts and (**e**) successfully detected animal.

Unfortunately, all videos have artifacts of random black and white lines across the frames or a higher than desired contrast of shimmering light on the water surface. In order to address these issues, a second step is taken after the ROI process is applied (see Figure 4a). This involves the use of Gaussian blur (see Figure 4b) in order to remove the aforementioned artifacts from the image caused by camera signal error and small waves on the surface of the water.

Once these steps are completed, a background frame is generated based on the weighted average of all the frames in the video. This results in the appearance of a frame that represents the pool, with the animal not yet present (see Figure 4c). The key step in the background subtraction-based method is to compare all the frames in the video to the generated background. In instances where there is a sufficiently large area of difference (see Figure 4d), this is where the animal will be in the video. As a last step, we need to find the optimal parameters of the bounding box for this area to use as the supposed position of the animal (see Figure 4e).

However, as anticipated, when tested on the complete set of videos, we find that in too many cases, the background subtraction-based recognition between the frames does not accurately find the position of the animal. Despite the preprocessing steps taken so far, the videos still contains cases where the test animal is challenging to recognize. In order to filter out these cases, we manually select and verify 50 videos, with 32,163 frames in total, as the training dataset for the neural network.

### 2.3. Animal Detection

In order to address the challenge of accurately recognizing the position of animals in videos, the previously mentioned manually curated training dataset is utilized to improve the accuracy of our animal detection system by training a convolutional neural network (CNN). By leveraging the power of deep learning, we aim to enhance the performance of our system and overcome the limitations observed in the background subtraction-based recognition method. One popular approach for image recognition discussed in the academic literature is the YOLOv5 architecture [12], a type of convolutional neural network. This method involves dividing the image into grid regions and making simultaneous predictions for the bounding boxes and classification results based on each region. One noteworthy benefit of this approach is its ability to detect specific objects in real-time, which makes it highly suitable for applications requiring the quick and accurate identification of objects within images or video streams. Additionally, the YOLOv5 architecture has shown promising results in various object detection tasks [13,14,15] making it a suitable choice for improving the accuracy of our animal detection system in videos.

Training a complex CNN takes a significant amount of time. Therefore, we use the YOLOv5m configuration of the pre-trained model and weights with 21.2 million parameters published by Ultralytics [12]. Initially, this model does not align directly with the requirements of our recognition problem. However, after fine-tuning it through 600 epochs of training using the tools included with the published source codes of YOLOv5 on our manually curated dataset, it can be tailored to address this specific challenge. This transfer-learning approach significantly reduces the time and quantity of data needed in comparison to training the entire CNN from scratch. Through this transfer-learning approach, we are able to leverage the knowledge and features learned from the pre-trained model and fine-tune them to improve the accuracy of the detections for the problem at hand.

This methodology is highly transferable since only the inner YOLOv5 model needs to be additionally trained on the new dataset. The set of videos that we process during this study includes two significantly different subsets, varying in the environmental lighting and the coloring of the animals. When the inner model is only trained on the first subset, its detection performance is not suitable for the other subset. Subsequent training of the model on data from the second subset results in a significant enhancement of its detection capabilities.

**Figure 5:**
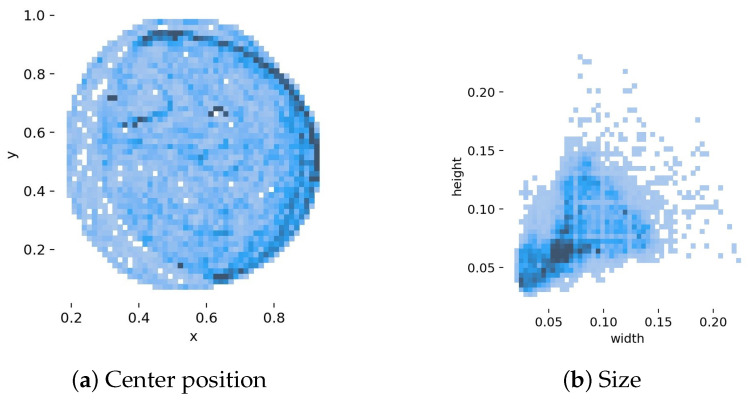
Dispersion of the normalized properties of the input bounding boxes from the training set: (**a**) center point positions and (**b**) dimensions.

The dataset is divided into a training and a validation set at a ratio of 4:1 to assess the network’s recognition accuracy during the learning process. This leads to 25,728 frames being assigned to the training set out of a total of 32,163 annotated frames, while 6435 frames are designated for the validation set. Figure 5 illustrates the key characteristics of the bounding boxes in the training set. The properties of these bounding boxes are between 0 and 1 since YOLOv5 only accepts input values that have been normalized to the dimensions of the input images. Panel (a) displays the center positions, while panel (b) highlights the variation in bounding box size.

To determine the trajectory of the swimming animal, it is essential to accurately track the animal’s position in each frame of the video. Therefore, before generating the swimming paths, the final step is to utilize the fine-tuned YOLOv5 for predicting the precise location of the animal in every video frame. This process yields individual files for each predicted frame that include the respective positions of the animal. In addition to the position files, post-detection videos are generated for each input video. These videos serve as a visualization of the image recognition process, allowing for the validation of the resulting data.

It is important to recognize that the identified trajectory of the animal may not always be precise, as YOLOv5 detection still has the potential for inaccuracies. As a result, it is essential to include a postprocessing stage to rectify any errors in the findings.

### 2.4. Postprocessing

**Figure 6:**
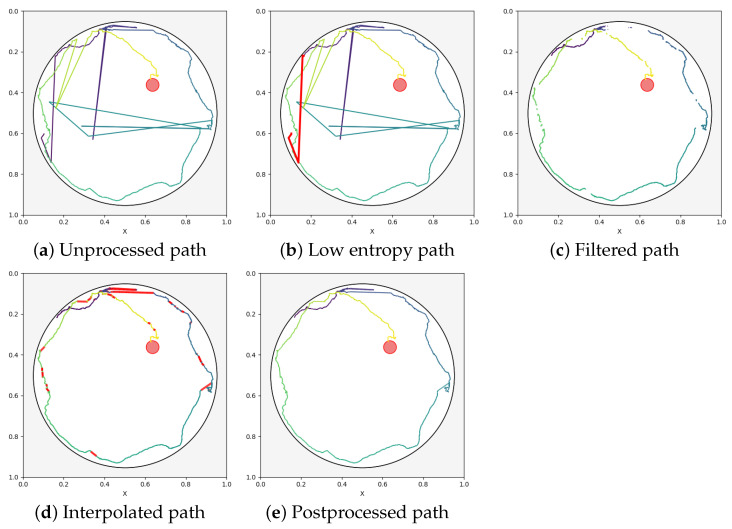
Path states (highlighted in red) during postprocessing: (**a**) unprocessed detected path from YOLOv5, (**b**) low-entropy discard at the start of the video, (**c**) filtering based on the 95th percentile, (**d**) interpolation where the segments are filtered out, and (**e**) the final postprocessed corrected path.

The process of constructing the swimming path (Figure 6) consists of a sequence of actions intended to precisely identify and assess inaccuracies in the observed movements of the experimental subjects. To begin with, this process entails creating continuous paths by combining positional data from each frame.

To ensure precision, a dynamic data cleaning process is utilized through continuous calculation of entropy within sliding windows. Frames with low entropy values (<0.1) indicating static sections in the videos are disregarded (see Figure 6b). Additionally, another filtering process is used to identify irregular movements based on the distance between the animal’s positions in consecutive frames. Anomalous positions exhibiting movement greater than the 95th percentile of all calculated distances are removed (see Figure 6c). Finally, any absent frames are linearly interpolated to ensure that the path remains continuous (see Figure 6d).

### 2.5. Feature Extraction

In this section, we explore the process of deriving characteristics from the swimming paths. As previously mentioned, analyzing the behavior and spatial learning abilities of animals through MWM tests can involve using a range of metrics. While some studies consider only swimming distance, escape latency, and swimming speed as key measures, a broader set of features could enhance the assessment of experiments comparing animal behavior according to research by [8,16].

In addition to utilizing the four quadrants of the pool as described in [3], we propose a novel method for dividing the pool into zones. This involves the use of concentric circles around the platform to create zone boundaries which allows us to experiment with smaller segments of the pool and thus evaluate the behavior of the animal at different distances from the platform.

**Figure 7:**
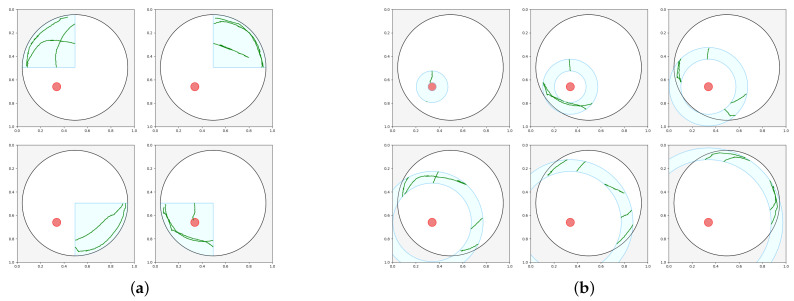
The different area-based segmentations: (**a**) the 4 quadrants, and (**b**) the concentric circles.

The first approach involves dividing the pool into four quadrants (refer to Figure 7a) and analyzing swimming paths within each quadrant, which is a commonly used technique in numerous MWM studies. In contrast, our novel approach utilized a predefined parameter to establish the width of concentric circles (see Figure 7b). Going forward, both the quadrant-based section and the concentric circle-based section of the pool will be referred to as a zone.

Furthermore, we carry out experiments using different widths of concentric circle-based sections, measured in terms of the platform diameter (D). This allows us to utilize smaller segments of the swimming paths for a more detailed analysis. The rationale for this approach is that key features are likely closely linked to how near the animal approaches the escape platform.

With the help of these two types of spatial components, we can define a wide range of derived features for the analysis of any given swimming path. For a comprehensive list of the 32 measures that are implemented for the evaluation process, please refer to Table 1.

Given that the actual placement of the platform may vary across experiments, we need some way to stay consistent with naming the different zones. To ensure consistency across the zone-specific measures, we select the first zone to always be the one containing the platform. For quadrants, this means that the first zone is where the platform is situated, and then we proceed in a clockwise direction. However, for concentric circles, we start with the innermost and move towards the outermost annulus, following the sequence as shown in Figure 7b.

**Figure 8:**
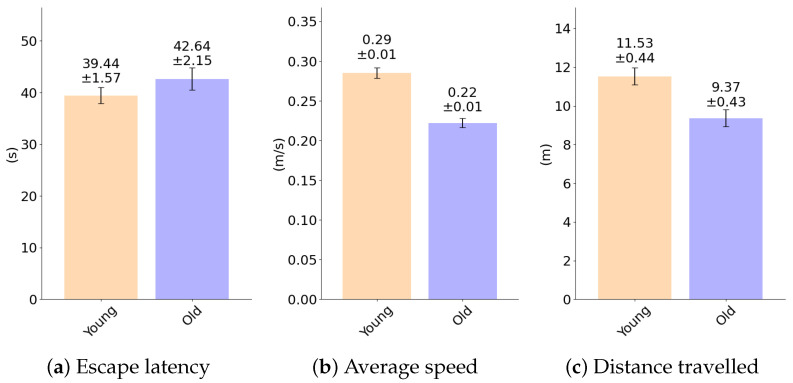
Mean and standard error of mean (SEM) values for (**a**) escape latency, (**b**) average speed, and (**c**) distance traveled.

To provide better interpretation for the differences between the two age groups of the rats, let us show in Figure 8 the mean and standard error of mean (SEM) values calculated for some key metrics, such as escape latency (a), average speed (b), and distance traveled (c). These metrics support our hypothesis that younger rats outperform the older rats during the tests: they are faster to escape, swim at higher average speeds, and cover more distance in less time.

### 2.6. Classification

In this study, we apply various widely used classification algorithms to classify our data points, including logistic regression (LR), Naive Bayes (NB), decision tree (DT), random forest (RF), and neural network (NN). We use this classification task as a tool to show how the novel concentric circle-based features provide additional relevant information compared to using only the quadrant-based features. Accordingly, this means that the accuracy scores are primarily intended for the purpose of comparison with each other. Hence, by incorporating the annuli-based metrics into the training data, and if the classification accuracy increases, we can assume that we have found useful new descriptors for animal behavior analysis. The target is to classify instances into one of the following two categories: old and young animals, encompassing a total of 251 old and 341 young cases. To validate the classification accuracy and avoid overfitting on the training data, we utilize 10-fold cross-validation. Thus, the dataset is partitioned into 10 equal subsets, with 9 of them used for training the model and the remaining used for validation. The training is then run 10 times, each time using a different subset for validation. The final metrics are obtained by averaging the results from these 10 iterations.

## 3. Results

In this section, we present the results of the classification models by showing the accuracy measures of the various methods tested with multiple different selections of the input data: (1) only including the number of entries and the time spent in the zones (Experiment 1), (2) working with all 32 features calculated on the quadrant-based zones exclusively (Experiment 2), (3) working with all 32 features calculated on the annulus-based zones exclusively (Experiment 3), (4) a combination of both (Experiment 4), and (5) selectively choosing the most informative features from the merged (from Experiment 4) feature list (Experiment 5). In Experiment 6, we test different values for the width of the annulus parameter for the concentric circles.

### 3.1. Experiment 1—Simple Features

In this first setup, we use only two measures: the number of entries into a zone by the animal and the time spent in that zone. Both of them are calculated for the four quadrant zones, producing eight different features as input fields for all five classification models. The performance of the five different classifiers can be evaluated by comparing their confusion matrices seen in Table 2, which shows how many times each classifier misidentified the animals’ age based on their behavior.

Table 3 shows the accuracy metric for all the classification models. It is evident that only utilizing these features might not be enough; thus, we refer to the literature to expand these features with commonly used metrics of animal behavior to create Experiment 2.

### 3.2. Experiment 2—Quadrant Features

In the first MWM tests conducted by [3], the measures of performance are the following: the escape latency, path length, path directionality, and the number of crossings between quadrant boundaries in the pool. In later studies, further measures are introduced, such as percentage of time spent in each quadrant [4] or more specifically the time spent in the target quadrant in comparison to other quadrants. Also, Dalm [7] lists the total distance covered in search of the platform, the time the animal spends near the wall, the average speed, the average distance of the animal from the center of the platform, etc. To harness the potential of the most well-known and commonly used measures of the animal’s performance during the MWM tests, we propose Experiment 2 to include all 32 features from Table 1 calculated for all four quadrants. The accuracy values produced by the models using this setup can be seen in Table 4.

### 3.3. Experiment 3—Concentric Circle Features

In direct comparison to the 32 features for the four quadrants used in the previous experiment, we test the same set of features but now calculated for the different concentric circle zones. The number of these zones are determined by how many has a region still inside the area of the pool as we are getting further away from the platform. In the case of Figure 7b, there are six such zones. The accuracy scores in Table 5 show the different classifiers’ performance trained on the features calculated for concentric circles with an annulus as wide as the platform in the pool.

### 3.4. Experiment 4—Combined Features

After Experiment 2 and 3, as the next step, we begin testing the combination of the two feature sets, and provide all 32 measures calculated for both the four quadrants and the concentric circles. Table 6 shows the accuracy of the different classifiers trained on the features calculated for both types of zone definitions, where—in line with the previous experiment—the concentric circles have an annulus as wide as the platform in the pool. One can easily see that almost all classifiers (with the exception of the NB model) are improved compared to the models using only one or the other type of zone defined features.

### 3.5. Experiment 5—Combined Features with Feature Selection

As there are 32 distinct measures for every quadrant and every annulus, 480 features in total for each MWM test, it is evident that we need to apply a dimensionality reduction strategy to decrease the number of features. Our main intention with implementing this feature selection step is to assess how the annuli-based metrics compare to the conventional quadrant-based metrics in terms of feature performance and descriptive power. If the annuli-based metrics are still present after the feature selection, we can assume that they carry meaningful information. We use supervised attribute filtering to select the most meaningful attributes and see which of them prove to be the most relevant. Then, we take the first *n* features in order of relevance and run the different classifiers on the selected f feature set. We apply the BestFirst attribute selection algorithm, which explores the space of subsets of attributes through a greedy hill-climbing approach enhanced by a backtracking mechanism. The degree of backtracking is determined by adjusting the number of allowed consecutive non-improving nodes, which finally influences the selection of the final set of the most informative attributes.

Table 7 presents a list of the 33 most relevant features, which contains only 18 unique measures. This is due to the fact that many of the measures are duplicated for several zones. Table 8 presents the corresponding accuracy scores: the DT classifier’s accuracy has minor improvements, the LR and NN classifiers show slightly less accurate results, and only the NB classifier has notably better accuracy. However, the RF classifier also shows an improvement over all previous accuracy scores with a value of 95.74%. As the values in Table 6 and Table 8 show minimal differences in accuracy, we can assume that the 33 selected features have comparable descriptive power to the original 480 features.

### 3.6. Experiment 6—Annulus Variation

For the final setup, we analyze the effect of changing the width parameter of the annulus of the concentric circles. We systematically vary the width of the annulus, with the default annulus being the same length as the diameter as the platform [D1], and exploring the alternative widths of 1.5, 2, and 2.5 times the diameter of the platform, shown as [D1.5, D2, D2.5], respectively. To create the training set, we still utilize the feature selection process as seen in Experiment 5, but the D parameter is set differently each time. All the corresponding accuracy scores can be seen in Table 9.

## 4. Discussion

We have developed an efficient, fully automated pipeline for animal detection, trajectory tracking, and classification. Our approach incorporates both quadrant and concentric circle-based segmentation of the pool. Our initial findings suggest that augmenting the original quadrant-based feature set with concentric circle-related features enhances classification performance. Using both types of zoning and extracting the diverse set of 32 features significantly broadens the scope of behavioral metrics compared to the traditional method. Classification experiments using various machine learning algorithms demonstrated that incorporating concentric circle features, combined with feature selection strategies, can enhance the differentiation between young and aged animal cohorts. This enhancement primarily reflects a difference in the resolution of the same underlying metrics rather than introducing a novel biological insight. Similarly, the original circular quadrant-based division method is based on mathematical and computational principles rather than biological considerations, making it challenging to explain the improvements of the concentric circle-based division from a biological perspective only.

Out of all the experiments done in the classification process, Experiment 2 can be considered state of the art; however, expanding the features from these and then selecting an appropriate subset proves to have better performance by 3.25–7.74% depending on the classifier, shown in Table 10.

As it can be seen from the results, our proposed approach outperforms conventional quadrant-based analysis, providing a more nuanced understanding of spatial learning behaviors and their changes due to aging or neurodegenerative conditions.

## 5. Conclusions

In this study, we introduced a comprehensive AI-driven framework for the automated analysis of MWM experiments, which is designed to enhance the accuracy and objectivity of behavioral assessments. The implementation of a machine learning-based automated system for MWM data analysis represents a significant advancement in the field of behavioral neuroscience. Traditional manual scoring methods of MWM experiments are inherently time-consuming and susceptible to variability, limiting their applicability for large-scale studies. The presented automated pipeline overcomes these challenges by providing a consistent and detailed analysis of animal behavior through machine learning techniques, particularly CNNs and feature-based classification approaches.

Our experiments reveal that combining quadrant and concentric circle-based features significantly improved the accuracy of distinguishing between younger and older animals, with random forest classifiers achieving the highest performance. The incorporation of concentric circle segmentation allowed for a more granular analysis of spatial learning strategies, particularly regarding proximity to the escape platform, which appears to be a critical factor in differentiating age-related cognitive differences. The feature selection further refined the models, reducing dimensionality while enhancing classification performance, thus underscoring the importance of targeted feature engineering.

The ability of our approach to reliably classify behavioral differences across age groups lays the foundation for its use in a broader range of neurodegenerative models, particularly Alzheimer’s disease. By improving both the efficiency and accuracy of data processing, this framework has the potential to facilitate new discoveries regarding the behavioral manifestations of cognitive decline and the efficacy of therapeutic interventions. However, future research should focus on expanding the dataset and exploring additional behavioral and swimming path-derived features related to the capabilities of the animals, such as path efficiency, a promising new comparison metric, that is already available in some tracking software. These could provide even deeper insights into the mechanisms underlying learning and memory. Additionally, applying this approach to rats with Alzheimer’s disease could help refine its utility in preclinical research and help explain the biological significance of the selected features and the novel pool sectioning method in behavior analysis. Furthermore, integrating this automated analysis with physiological or molecular data could offer a more comprehensive understanding of the neurobiological basis of the observed behaviors.

## Figures and Tables

**Table 1:** List of all 32 measures that are implemented for the evaluation process.

Feature Name [Unit of Measurement]			
1.	Absolute turn angle while in zone [°]	17.	Longest visit to zone [s]
2.	Average absolute heading error to platform [°]	18.	Maximum distance to zone border [m]
3.	Average absolute heading error to zone [°]	19.	Maximum distance to zone center [m]
4.	Average distance to zone border [m]	20.	Maximum speed in zone [m/s]
5.	Average distance to zone center [m]	21.	Minimum distance to zone border [m]
6.	Average duration of visit [s]	22.	Minimum distance to zone center [m]
7.	Average speed [m/s]	23.	Number of entries to zone
8.	Cumulative distance from zone [m]	24.	Number of exits from zone
9.	Distance traveled in zone [m]	25.	Shortest visit to zone [s]
10.	Distance traveled until first entry [m]	26.	Signed initial heading error to platform and zone center [°]
11.	Initial distance to zone [m]	27.	Starting zone
12.	Initial heading error to platform and zone center [°]	28.	Time moving away from zone [s]
13.	Latency of first entry to zone [s]	29.	Time moving towards zone [s]
14.	Latency of first exit from zone [s]	30.	Time oriented towards platform while in zone [s]
15.	Latency of last entry to zone [s]	31.	Time oriented towards zone center while in zone [s]
16.	Line crossings	32.	Total time in zone [s]

**Table 2:** Confusion matrices of the classifiers for Experiment 1.

LR				NB				DT			
		Predicted				Predicted				Predicted	
		Young	Old			Young	Old			Young	Old
True	Young	131	112	True	Young	124	119	True	Young	132	111
	Old	66	275		Old	77	264		Old	118	223
		**RF**				**NN**					
				Predicted				Predicted			
				Young	Old			Young	Old		
		True	Young	141	102	True	Young	151	92		
			Old	88	253		Old	81	260		

**Table 3:** Accuracy metrics of the classification methods: logistic regression, Naive Bayes, decision tree, random forest, and neural network for Experiment 1.

Experiment	Accuracy of the Classifiers				
	LR	NB	DT	RF	NN
Number of entries and time spent in zones	0.6952	0.6643	0.6077	0.6745	0.7036

**Table 4:** Accuracy metrics of the classification methods: logistic regression, Naive Bayes, decision tree, random forest, and neural network for Experiment 2.

Experiment	Accuracy of the Classifiers				
	LR	NB	DT	RF	NN
All 32 features calculated on quadrants	0.7534	0.7688	0.8374	0.9315	0.7568

**Table 5:** Accuracy metrics of the classification methods: logistic regression, Naive Bayes, decision tree, random forest, and neural network for Experiment 3.

Experiment	Accuracy of the Classifiers				
	LR	NB	DT	RF	NN
All 32 features calculated on annuli	0.7653	0.7296	0.7913	0.8718	0.8152

**Table 6:** Accuracy metrics of the classification methods: logistic regression, Naive Bayes, decision tree, random forest, and neural network for Experiment 4.

Experiment	Accuracy of the Classifiers				
	LR	NB	DT	RF	NN
All 32 features calculated on quadrants and annuli	0.8184	0.7639	0.8598	0.9435	0.8168

**Table 7:** The best performing features after feature selection.

1.	Quadr. 1—Maximum distance to zone border	18.	Ann. 1—Average absolute heading error to platform
2.	Quadr. 1—Maximum distance to zone center	19.	Ann. 1—Average absolute heading error to zone
3.	Quadr. 1—Number of entries to zone	20.	Ann. 1—Average distance to zone center
4.	Quadr. 2—Average absolute heading error to zone	21.	Ann. 1—Initial heading error to platform and zone center
5.	Quadr. 2—Average distance to zone center	22.	Ann. 1—Signed init. heading err. to plat. and zone center
6.	Quadr. 2—Maximum distance to zone border	23.	Ann. 1—Total time in zone
7.	Quadr. 2—Time oriented towards platform while in zone	24.	Ann. 2—Average speed
8.	Quadr. 2—Latency of first exit from zone	25.	Ann. 3—Average duration of visit
9.	Quadr. 3—Average absolute heading error to platform	26.	Ann. 3—Maximum speed in zone
10.	Quadr. 3—Maximum distance to zone border	27.	Ann. 5—Average absolute heading error to platform
11.	Quadr. 3—Maximum speed in zone	28.	Ann. 5—Maximum distance to zone border
12.	Quadr. 3—Total time in zone	29.	Ann. 5—Longest visit to zone
13.	Quadr. 4—Absolute turn angle while in zone	30.	Ann. 6—Maximum distance to zone border
14.	Quadr. 4—Average distance to zone border	31.	Ann. 6—Average speed
15.	Quadr. 4—Maximum distance to zone border	32.	Ann. 7—Absolute turn angle while in zone
16.	Quadr. 4—Distance travelled in zone	33.	Ann. 7—Maximum distance to zone border
17.	Quadr. 4—Time oriented towards platform while in zone		

**Table 8:** Accuracy metrics of the classification methods: logistic regression, Naive Bayes, decision tree, random forest, and neural network for Experiment 5.

Experiment	Accuracy of the Classifiers				
	LR	NB	DT	RF	NN
Experiment 5 with feature selection	0.7928	0.8116	0.8700	**0.9574**	0.7930

**Table 9:** Accuracy metrics of the classification methods: logistic regression, Naive Bayes, decision tree, random forest, and neural network for Experiment 6.

Experiment	Annuli Width (D)	Accuracy of the Classifiers				
		LR	NB	DT	RF	NN
Experiment 6 with varying annuli	D1	0.7928	0.8116	0.8700	**0.9574**	0.7930
	D1.5	0.8134	0.7998	0.8905	**0.9470**	0.7706
	D2	0.8308	0.7859	0.8665	**0.9437**	0.8186
	D2.5	0.7740	0.7826	0.8973	**0.9640**	0.7997

**Table 10:** Improved accuracy metrics for each used classification method, comparing Experiment 2 to Experiment 6.

	Accuracy of the Classifiers				
	LR	NB	DT	RF	NN
Experiment 2	0.7534	0.7688	0.8374	0.9315	0.7568
Best of Experiment 6 (annulus width)	0.8308 (D2)	0.8116 (D1)	0.8973 (D2.5)	0.9640 (D2.5)	0.8186 (D2)
**Improvement**	**0.0774**	**0.0428**	**0.0599**	**0.0325**	**0.0618**

## Data Availability

The raw data supporting the conclusions of this article will be made available by the authors on request.

## Abbreviations

The following abbreviations are used in this manuscript:

CNS	Central nervous system
CA1-CA3	Cornus Ammonis-1—Cornus Ammonis-3
CNN	Convolutional neural network
DT	Decision tree
LR	Linear regression
MDPI	Multidisciplinary Digital Publishing Institute
MWM	Morris Water Maze
NB	Naive Bayes
NN	Neural network
RF	Random forest
ROI	Region of Interest
